# Genetic Regulation of Bone Metabolism in the Chicken: Similarities and Differences to Mammalian Systems

**DOI:** 10.1371/journal.pgen.1005250

**Published:** 2015-05-29

**Authors:** Martin Johnsson, Kenneth B. Jonsson, Leif Andersson, Per Jensen, Dominic Wright

**Affiliations:** 1 AVIAN Behavioural Genomics and Physiology group, IFM Biology, Department of Physics, Chemistry and Biology, Linköping University, Linköping, Sweden; 2 Department of Surgical Sciences, Orthopaedics, Akademiska Sjukhuset, Uppsala University, Uppsala, Sweden; 3 Department of Medical Biochemistry and Microbiology, BMC, Uppsala University, Uppsala, Sweden; The University of North Carolina at Chapel Hill, United States of America

## Abstract

Birds have a unique bone physiology, due to the demands placed on them through egg production. In particular their medullary bone serves as a source of calcium for eggshell production during lay and undergoes continuous and rapid remodelling. We take advantage of the fact that bone traits have diverged massively during chicken domestication to map the genetic basis of bone metabolism in the chicken. We performed a quantitative trait locus (QTL) and expression QTL (eQTL) mapping study in an advanced intercross based on Red Junglefowl (the wild progenitor of the modern domestic chicken) and White Leghorn chickens. We measured femoral bone traits in 456 chickens by peripheral computerised tomography and femoral gene expression in a subset of 125 females from the cross with microarrays. This resulted in 25 loci for female bone traits, 26 loci for male bone traits and 6318 local eQTL loci. We then overlapped bone and gene expression loci, before checking for an association between gene expression and trait values to identify candidate quantitative trait genes for bone traits. A handful of our candidates have been previously associated with bone traits in mice, but our results also implicate unexpected and largely unknown genes in bone metabolism. In summary, by utilising the unique bone metabolism of an avian species, we have identified a number of candidate genes affecting bone allocation and metabolism. These findings can have ramifications not only for the understanding of bone metabolism genetics in general, but could also be used as a potential model for osteoporosis as well as revealing new aspects of vertebrate bone regulation or features that distinguish avian and mammalian bone.

## Author Summary

In this work we seek to further the understanding of bone genetics by mapping bone traits and gene expression in the chicken. Bone in female birds is special due to egg production. In this study, we combine the genetic mapping of bone traits with bone gene expression to find candidate quantitative trait genes that explain the differences between wild and domestic chickens in terms of bone production. The concept of combining genetic mapping and gene expression mapping is not new, and has already been successful in isolating bone-related genes in mammals, however this is the first time it has been applied to an avian system with such unique bone modelling processes. We aim to reveal new molecular mechanisms of bone regulation, and many of the candidates we find are new, highlighting the potential this technique has to identify the potential differences between avian and mammalian bone biology.

## Introduction

The genetic basis of bone mineral density in the chicken is of both theoretical and practical importance. On the theoretical side, domestication is an example of intense directional selection causing wide-ranging differences in the phenotype of domestic animals compared to their wild progenitors. In the case of the domestic chicken, descended from the wild Red Junglefowl, bone metabolism is intrinsically coupled with the production of eggs. Hence, bone and fecundity phenotypes form a suite of correlated traits that have likely been tied with fitness during domestication. In addition, the comb, a sexual ornament in both male and female chickens [1,2], appears to be tied with bone mineral density. In terms of practical applications, bone mineral density is a predictor of osteoporosis, a debilitating condition in both chickens [3,4] and humans [5]. Describing quantitative trait genes for bone mineral density can give new clues about the biological processes underlying osteoporosis. Also, to the extent that the variants are still segregating in commercial chicken flocks, they may provide targets for selection.

Bone is made up of a mineral matrix, mostly hydroxyapatite, in an organic matrix dominated by collagen, lipids, proteoglycans and other bone structural proteins. Bone is continually being remodelled by osteoblasts, which produce bone components, and osteoclasts, which break them down. Bone metabolism in female birds is special in that they produce a medullary bone, which serves as a reservoir for calcium used in production of the eggshell [6,7]. Avian bone remodelling is quicker than mammalian and coordinated with the laying cycle [8,9]. Osteoporosis, loss of bone density, and bone fractures are a major health issue for layer chickens in production, likely exacerbated by the strains that high egg production and quick growth put on domestic chickens [10]. There is substantial genetic variation in bone traits in domestic layer chickens [11–13]. Studies involving dietary treatments suggest that the cause of osteoporosis in hens is not merely calcium deficiency [13,14], and that there is a genetic basis to bone strength independent of calcium metabolism.

With osteoporosis also being a serious health problem in humans, genetic mapping studies in humans and animal models have been performed to search for genes and processes involved in bone density. As is the case with heritable quantitative traits in general, the bone mineral density loci that have been identified so far leave most of the genetic variance unexplained (see review by [15]). Linkage mapping rarely has the resolution to isolate single molecular genes, but linkage mapping in humans has found variants affecting bone density in genes such as *LRP5* [16–18] and *BMP2* [19]. Genome-wide association studies, on the other hand, bring associated regions down to few or sometimes a single gene. In recent years, several such association studies have been published, implicating common genetic variants in human bone density variation. The hits include known bone-related genes such as osteoblast regulator osterix, osteoclast regulators *RANKL* and osteoprotegerin, and Wnt pathway genes such as β-catenin and *WNT16* [20–31]. However, even with the high resolution of a GWAS, it is not necessarily the gene closest to the variant that is the causal gene. Authors have successfully employed eQTL mapping or genetical genomics and systems genetics approaches such as coexpression networks, to isolate quantitative trait genes for bone density in mice, including *Alox5* [32], *Alox15* [33], *Bicc1* [34], *Asxl2* [35] and *Darc* [36].

Our aim is to study the genetics of bone traits in the chicken using modern genomics methods. Due to the divergence of domestic chickens from Red Junglefowl during domestication, we can use a wild by domestic chicken cross as a study system for bone genetics. Quantitative trait locus (QTL) mapping as a top down mapping approach has been widely successful when it comes to finding chromosomal regions associated with various traits. However, going from the quantitative trait locus, usually a broad chromosomal region harbouring many genes, to causative genes or even variants, is difficult. The main obstacle to quantitative trait gene identification is the long linkage blocks in the pedigrees employed for mapping experiments. Making an advanced intercross line is one strategy to increase informative meioses and mapping resolution [37]. The chicken has a high recombination rate and a relatively compact genome compared other vertebrate models such as the mouse [38].

To aid quantitative trait gene identification, we turn to genetical genomics or expression QTL (eQTL) mapping to female femoral gene expression measured by microarrays. While gene regulation is complex, regulatory variation in molecular intermediates can be assumed to have simpler genetic architecture than the phenotypic trait, which is affected by the combined output of many such molecular pathways. In cases where the causative variant acts by means of gene expression, expression QTL mapping helps by reducing the number of candidate genes to the ones whose expression map to the QTL region. Beyond overlapping bone QTL and expression QTL, we consider the regression between bone trait and gene expression level of these positional candidate genes. In this way, we only retain candidates whose gene expression is associated with the bone trait.

## Results

**Fig 1:**
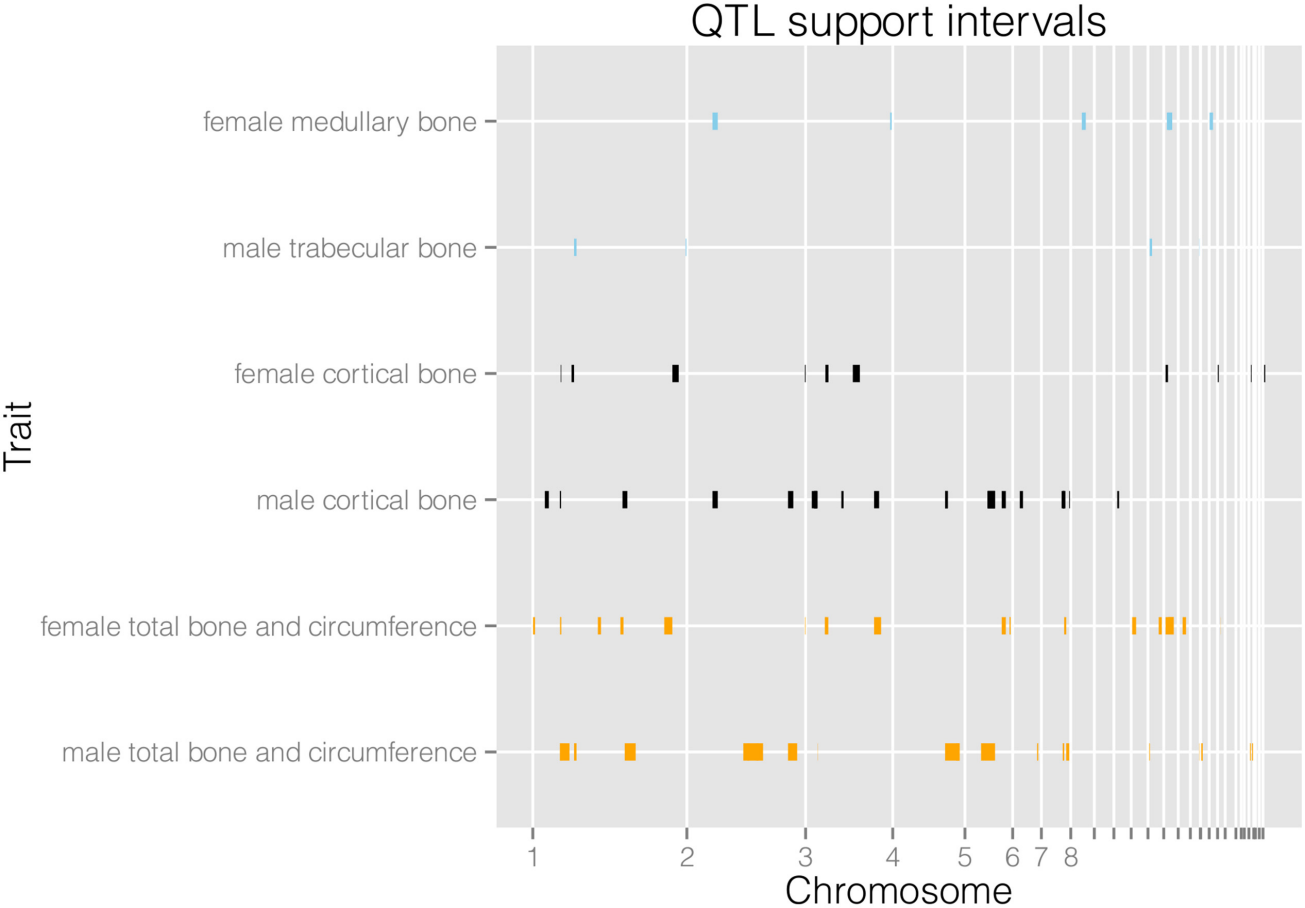
Physical locations of QTL support intervals. We display medullary, cortical and total area and circumference related traits for males and females separately. Their architectures are largely separate, with a few regions of overlap.

We find a total of 94 QTL for 21 traits (see Table 1 and S1 Table). When we combine loci for different variables they form 25 separate genomic regions associated with female bone traits, and 26 separate regions for male bone traits. We detect a considerable amount of digenic epistasis in the form of 40 epistatic pairs. As can be seen in Fig 1, the female and male QTL are largely separate. Additionally, medullary traits in females and trabecular traits in males are largely distinct from the cortical and total bone traits. We compare the results with previously published QTL from the F_2_ generation of this intercross. The advanced intercross (AIL) female and male QTL together form 41 genomic intervals, while the F_2_ QTL form 19 intervals. There are 11 regions of overlap between the sets on chromosomes 1, 2, 3, 6, 7, 13, and 18 (S6 Table). Variances explained by individual QTL and full QTL models including epistasis are given in S1 and S7 Tables.

**Table 1:** Table of high-confidence candidate eQTL with location of the gene, LOD score, and p-value for association between gene expression and bone trait.

Trait	Gene	Chromosome	Position (Mb)	eQTL LOD	p-value for association
diaphyseal total density	GNS	1	36	3.8	0.00018
diaphyseal endosteal circumference	GNS	1	36	3.8	0.00161
diaphyseal cortical density	603961757F1	1	37	5.6	0.00198
diaphyseal cortical density	uncharacterized gene	1	37	4.6	0.00060
diaphyseal cortical area	TSTA3	2	155	3.5	0.00030
diaphysal total content	KHDRBS3	2	149	4.0	0.00005
diaphysal total content	TSTA3	2	155	3.5	0.00001
diaphysal total content	PTK2	2	151	8.0	0.00247
diaphyseal cortical thickness	KHDRBS3	2	149	4.0	0.00001
diaphyseal cortical thickness	TSTA3	2	155	3.5	0.00037
diaphyseal cortical thickness	MRPS18A	3	32	2.5	0.00039
diaphyseal cortical thickness	uncharacterized gene	3	32	8.2	0.00165
diaphyseal endosteal circumference	CENPO	3	108	2.9	0.00019
diaphyseal medullary area	603847051F1	3	99	3.0	0.00000
diaphyseal medullary area	603846396F1	3	113	6.2	0.00050
diaphyseal medullary area	603961442F1	3	113	6.7	0.00053
diaphyseal medullary content	COL11A1	8	12	2.7	0.00024
diaphyseal total area	RAB24	13	10	4.2	0.00027
diaphyseal total area	SIMC1	13	10	4.3	0.00122
diaphyseal medullary area	DBN1	13	10	2.8	0.00301
metaphyseal total content	B4GALT7	13	10	10.0	0.00342
metaphyseal total content	osteonectin	13	13	2.8	0.00026
diaphyseal cortical thickness	HSF5	19	1	4.3	0.00035
diaphyseal cortical thickness	ISGF9B	24	2	3.4	0.00069
diaphyseal cortical thickness	POU2AF1	24	4	2.6	0.00414

**Fig 2:**
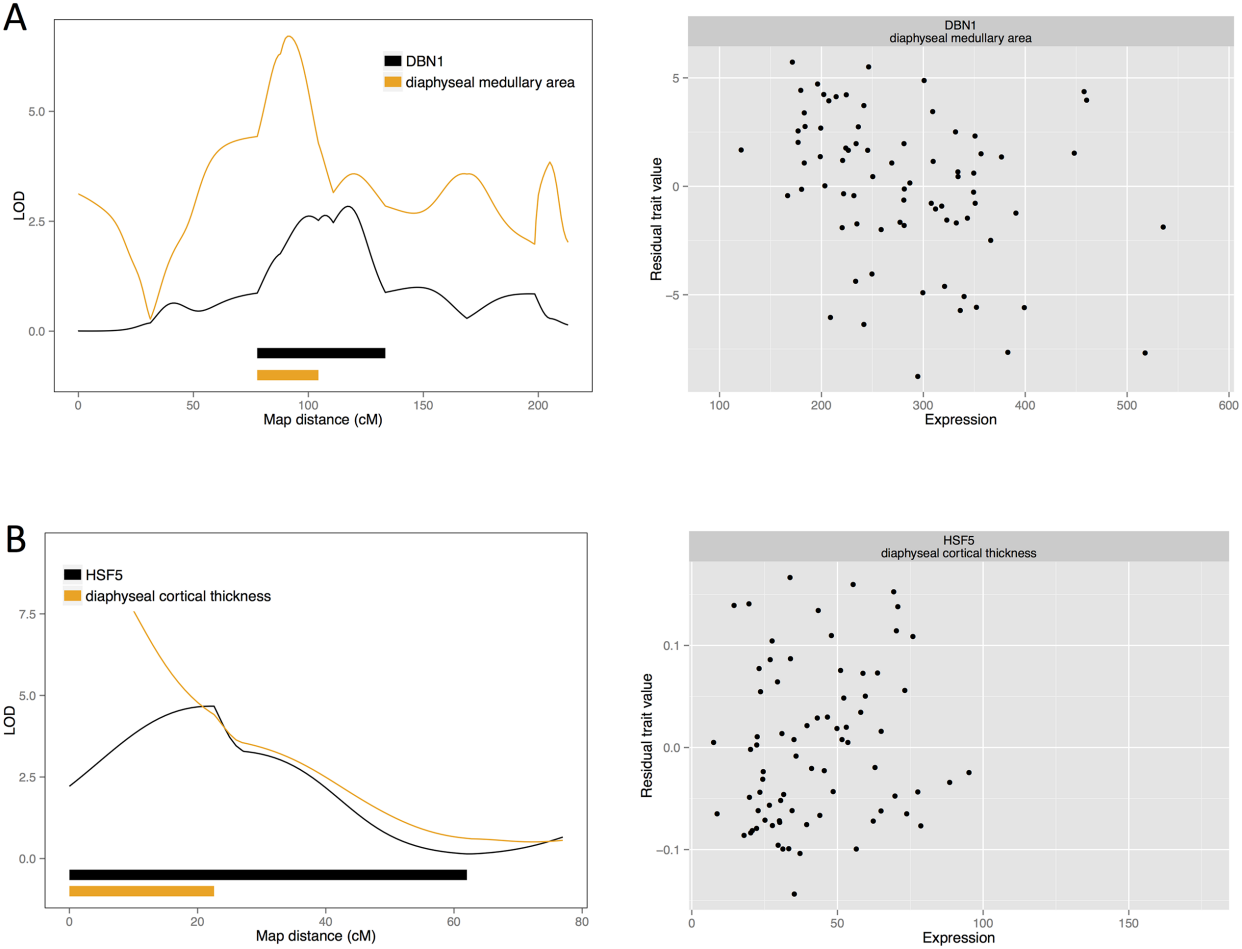
Candidate quantitative trait genes (A) *DNB1* for female medullary and (B) *HSF5* for female cortical traits: LOD curves and confidence intervals of bone QTL and associated eQTL, and scatterplots of residual phenotypes against gene expression values.

**Fig 3:**
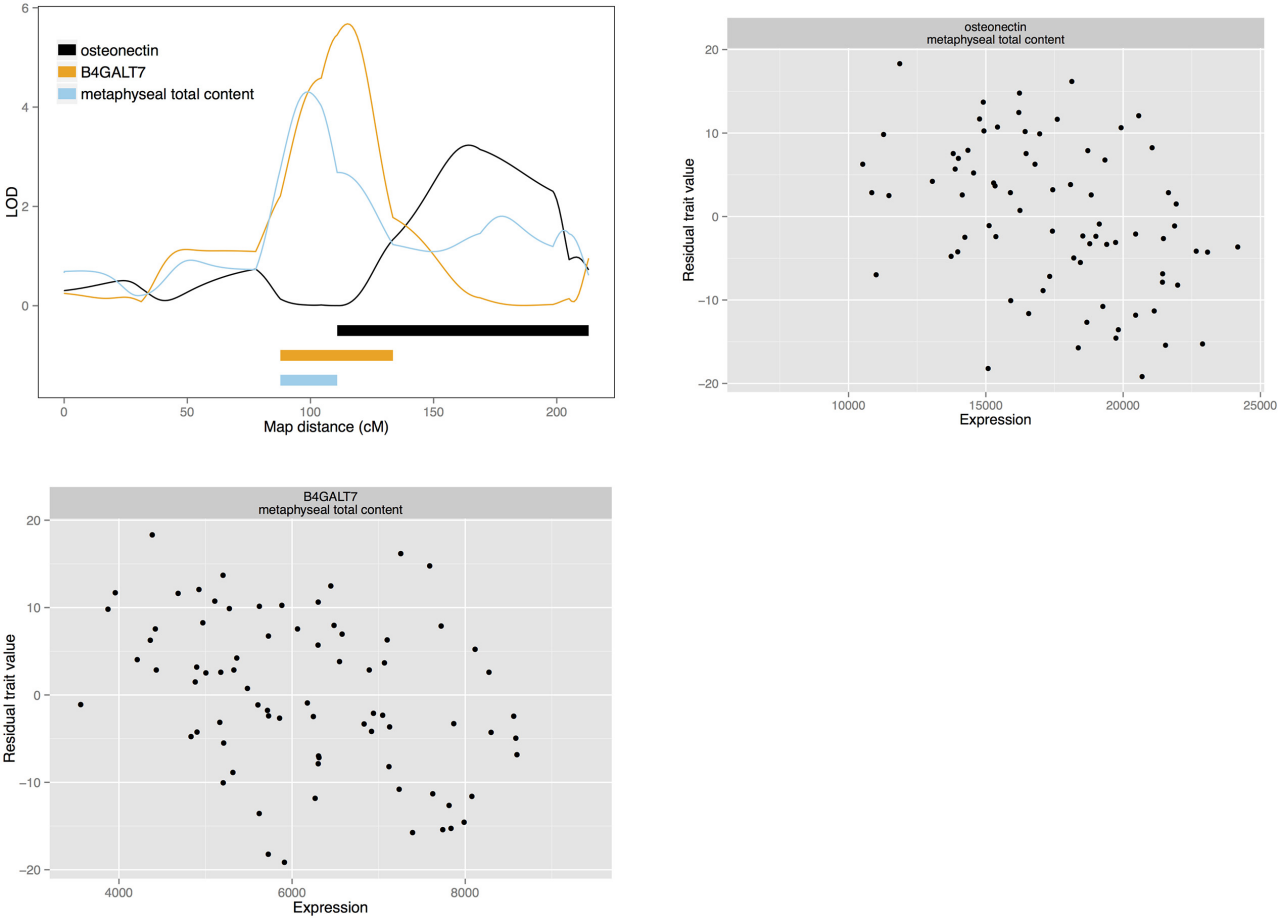
Candidate quantitative trait genes osteonectin and *B4GALT7* for total bone content: LOD curves and confidence intervals of bone QTL and associated eQTL, and scatterplots of residual phenotypes against gene expression values.

We detect 6318 local cis-eQTL (see S3 Table) and 1470 distal trans-eQTL that affect female femoral gene expression. The eQTL study reveals a copious amount of gene-regulatory variation, but only a small subset of eQTL will be relevant for the phenotypic QTL that we detected. Therefore we find those local eQTL confidence intervals that overlap the confidence intervals of the female bone QTL and test for an association between gene expression level and trait value. In light of the difference between male and female genetic architecture, we do not consider the male QTL (gene expression only being measured in female bone samples). A total of 71 genes are candidates for bone QTL in the sense that they have a local eQTL that overlaps a bone QTL and that their expression is associated with the trait. In this way, we find 15 candidates for medullary traits, 20 for cortical traits, and 45 for total content, circumference and area traits. While it is possible that a QTL effect is actually made up of several linked variants, we have higher confidence in a candidate if there are few alternative candidates with significant associations in the same QTL region. The complete list of associations is shown in S2 Table, and here we outline the high confidence candidates (Figs 2 and 3, S1 and S2 Figs).

### Candidate genes: Medullary traits

Beginning with the medullary traits, we find two candidate causal genes, each for a different QTL. These genes are *drebrin (DBN1; NM_205499)* for diaphyseal medullary area on chromosome 13, and *collagen type XI alpha 1* (*COL11A1*; represented by the EST sequence *603372847F1*) for diaphyseal medullary content on chromosome 8. Additionally, a QTL for diaphyseal medullary area on chromosome had three candidates: EST probesets *603847051F1*, *603961442F1* and *603846396F1*. These correspond to two unknown cDNA clones.

### Candidate genes: Cortical traits

There are three cortical trait QTL with a single candidate each. *Heat shock transcription factor family member 5 (HSF5; ENSGALG00000001031)* is a candidate for diaphyseal cortical thickness on chromosome 19. Also, *tissue specific transplantation antigen P35B (TST3A; ENSGALG00000016129)* is a candidate for diaphyseal cortical area on chromosome 2. This QTL colocalises with another for diaphyseal cortical thickness, which has both *TST3A* and *KH domain-containing*, *RNA-binding*, *signal transduction-associated protein 3 (KHDRBS3; ENSGALG00000016203)* as candidates. *Mitochondrial ribosomal protein S18A (MRPS18A; ENSGALG00000010296)* and an uncharacterized gene from the Ensembl database (*ENSGALG00000010303)* are candidates for diaphyseal cortical thickness on chromosome 3. *Immunoglobulin superfamily*, *member 9B (IGSF9B; ENSGALG00000001450)* and *POU class 2 associating factor 1 (POU2AF1; NM_204175)* are also candidates for a diaphyseal cortical thickness QTL on chromosome 24. Finally, on chromosome 1 we find an uncharacterized Ensembl gene *ENSGALG00000021181* and EST probeset *603234378F1* as candidates for diaphyseal cortical density. However, both these probesets correspond to the predicted gene *LOC771935 (XM_001235144*.*2)*, which has since been removed from GenBank.

### Candidate genes: Total bone and circumference

We also detect gene expression candidates for total bone and bone circumference. *Glucosamine (N-acetyl)-6-sulfatase (GNS; NM_001199559)* is the sole candidate for a diaphyseal total bone density and a diaphyseal endosteal circumference QTL on chromosome 1. Also, *centromere protein O (CENPO)*, represented by EST *603256840F1*, is a candidate for a diaphyseal endosteal circumference QTL on chromosome 3. On chromosome 13, *secreted protein*, *acidic*, *cysteine-rich (osteonecin)* represented by the EST probeset *603470410F1* and *xylosylprotein beta 1*,*4-galactosyltransferase*, *polypeptide 7 (B4GALT7; NM_001039911)* are candidates for a metaphyseal total content QTL. This QTL colocalises with the metaphyseal medullary area QTL for which *drebrin* is a candidate. *B4GALT7* and *osteonectin* are also among the candidates for a third, colocalising medullary content QTL. These genes may affect both total bone content and medullary content. In a similar fashion, *TSTA3*, *KHDRBS3* are candidates for diaphyseal total bone content, as well as the cortical traits mentioned above. However, in the case of diaphyseal total content, the QTL also has a third candidate: *protein tyrosine kinase 2 (PTK2)*, represented by EST probeset *603469577F1*. We also detect *Ras-related protein Rab-24 (RAB24)* and *SUMO-interacting motifs containing 1 (SIMC1)* as candidates for diaphyseal total area on chromosome 13.

### Translational comparisons

Finally, we compared our candidate quantitative trait genes with results from genome-wide association studies (GWAS) in mice and humans. 30 out of 76 candidate probesets could be mapped to a mouse ortholog. Three of them were associated with bone mineral density in the GWAS by Farber et al. [35]. These genes were *somatostatin receptor 5 (SSTR5; NM_001024834)*, *calcium channel*, *voltage-dependent*, *T type*, *alpha 1H subunit (CACNA1H; ENSGALG00000005215)*, *mitochondrial ribosomal protein S18A (MRPS18A)*, and *glycoprotein 1b*, *alpha polypeptide (GP1BA; ENSGALG00000021693)*. These genes are located in QTL that have other associated candidates, but these previous associations increase our confidence in them as potential causative genes. We also searched for our candidates among the top associated genes for bone mineral density in the dbGAP Association Results Browser, and found no overlap.

### eQTL hotspots

Hotspots of trans-eQTL occurring in the same region could reflect regulatory variants with many downstream effects. We searched for such hotspots by comparing the number of overlapping trans-eQTL with the number of trans-eQTL observed with simulated uniformly distributed eQTL intervals. In this way we found 12 clusters of trans-eQTL, making up putative hotspots on chromosomes 1, 2, 3, 4, 5 and 25 (see S4 Table). Four of these hotspots overlap female bone QTL. In particular, the hotspot on chromosome 3 overlaps the cortical thickness QTL for which we detect candidate genes *MRPS18A* and *ENSGALG00000010303*. Further dissection of these loci would be necessary to know whether the clusters are genuine trans-regulatory hotspots involved in bone function.

## Discussion

We report here QTL mapping and genetical genomics of several aspects of chicken bone density under domestication. These QTL explain part of the difference in these traits between wild and domestic chickens. We map QTL affecting total femoral bone, cortical bone and medullary bone, which is particular to laying hens. We find 25 genomic regions in total for female traits and 26 for male traits. Also, we identify 76 candidate genes correlated with female bone traits. A handful of these are high confidence candidates for quantitative trait genes acting by changes in gene expression.

In this work, we have combined quantitative trait locus mapping and genetical genomics to illuminate the genetic underpinnings of changes in bone deposition under chicken domestication. To our knowledge this is the first eQTL study performed on bone tissue using the chicken as a model. Although our study is reasonably large, both for a phenotypic QTL scan and an eQTL scan, genetic mapping is always limited by power. Complex traits are often massively polygenic and gene-regulatory effects, particularly in trans, can be subtle. In addition, how large an effect a QTL needs to have to be relevant for domestication and how large mediating eQTL effects have to be, are open questions. We find a genetic architecture of bone with several moderately large QTL and digenic epistasis. The eQTL mapping reveals a rich landscape of expression QTL, both local cis- and distal trans-acting. Though most of these eQTL are likely to be inconsequential for bone phenotypes, this demonstrates wide ranging gene-regulatory evolution during chicken domestication.

### Medullary bone candidates

Drebrin (*DBN1*) is the sole candidate for one of our medullary QTL. Drebrin binds actin filaments and is known for its role in the nervous system. However, it is also expressed in lymphocytes and involved in connecting chemokine receptor *CXCR4* to the cytoskeleton [39]. This receptor, in turn, is involved in differentiation of osteoclasts from hematopoietic cells [40]. While drebrin has not been studied in connection with bone metabolism, we hypothesise that eQTL effects on drebrin expression could affect medullary bone through osteoclasts. We also detect *collagen XI alpha 1* as a candidate for medullary content on chromosome 13 with multiple sources indicating effects on bone. Mutations in this gene cause the rare human chondrodysplasias fibrochondrogenesis [41], Marshall syndrome and Stickler syndrome [42,43], and may predispose to osteoarthritis [44]. Cell culture experiments suggest that it inhibits osteoblast differentiation [45].

### Cortical bone candidates

We find *HSF5* to be associated with cortical thickness. Heat shock factors (HSF) regulate heat shock protein (HSP) genes and activate them in response to cellular stressors. Furthermore, there is some evidence that heat shock responses may be involved in bone formation. Bone growth can be stimulated by heat, and heat treatment increases mineralisation and heat shock protein expression in cell culture [46]. Molecular studies of the *RANKL* promoter and *HSF2* knock-down suggest that this osteoclast regulator is regulated by heat shock factors [47,48]. However, there is little known about *HSF5* specifically. *TSTA3*, our candidate for a diaphyseal cortical area QTL, is involved in protein glycosylation by contributing to the synthesis of GDP-fucose, which is used by fucosyltransferases in glycosylation of cell adhesion proteins. Knockout studies of *TSTA3* in mice suggest that fucosylation is needed to regulate myeloid cell differentiation by Notch signalling [49]. Notch also regulates both osteoblast and osteoclast differentiation [50,51]. There are two additional candidates for the colocalising cortical thickness QTL: *KHDRBS3*, which encodes an RNA binding protein, and *PTK2*, protein tyrosine kinase. *PTK2* is involved in osteoblast differentiation [52,53]. Along with the unknown Ensembl gene, *ENSGALG00000010303*, *MRPS18A* is a candidate for diaphyseal cortical thickness, whilst *MPRS18A* encodes a mitochondrial ribosomal protein. These ribosomes translate proteins required for mitochondrial function. Hence mutations in mitochondrial ribosomal proteins can cause oxidative phosphorylation deficiencies (such as OMIM phenotypes 614582, 611719, 610498) [54]. Such defects can affect diverse organ systems, since mitochondrial function is crucial for cellular metabolism. To our knowledge, no such disorders with specific bone-related symptoms are known, although there is still the possibility of mitochondrial effects on bone. Active osteoclasts, for instance, are rich in mitochondria [55]. Quantitative changes in mitochondrial activity could influence the balance of bone remodelling. Finally, we find two candidates for a cortical thickness locus on chromosome 24: *IGSF9B* and *POU2AF1*. There is little known about *IGSF9B*, except that it is involved in cell adhesion at synapses [56]. *POU2AF1*, however, is known as a transcription factor in B cells of the immune system. It regulates B cell maturation, and is mutated in some forms of leukaemia [57,58]. While no direct connection between *POU2AF1* expression and bone is known, the immune system affects bone regulation. For example, B cells produce cytokines with effects on bone, including osteoclast-stimulating *RANKL* [59–61].

### Total bone and circumference candidates

*GNS* is our single candidate for a couple of colocalising QTL for diaphyseal total bone and endosteal circumference on chromosome 1. It encodes a glucosamine sulfatase that breaks down heparane sulphate. Heparan sulphates are polysaccharides that make up part of the extracellular matrix and are part of proteoglycan glycoproteins. As such, heparan sulphates have regulatory functions in development, including regulation of osteoblasts [62,63]. Mutations in the GNS gene cause mucopolysaccharidosis type IIID (Sanfilippo syndrome D; OMIM phenotype 252940) [64,65]. It is a lysosomal enzyme, and loss of function causes accumulation of heparan sulphate in the organelle with deleterious effects on several organ systems. Symptoms includes effects on bone, though these may be secondary effects [66]. Taken together, this leads to the speculation that the *GNS* eQTL may affect bone density through either a signaling effect of heparan sulphate or through a lysosome-dependent mechanism. *CENPO*, a candidate for an endosteal circumference QTL, encodes a centromere protein and is involved in chromosomal segregation during mitosis [67]. While proliferation of different bone cell populations is important for bone metabolism, its involvement in bone is unknown. Another locus for total bone content has two candidates: *osteonectin* and *B4GALT7*. *Osteonectin* is a glycoprotein produced by osteoblasts. It contributes to mineralisation by binding mineral crystals and linking to collagen, and it functions in bone remodelling [68,69]. *B4GALT7* is another candidate involved in protein glycosylation, a glycosyltransferase that participates in synthesis of proteoglycans, and in particular heparane sulphate. Mutations in human cause Ehler—Danlos syndrome (OMIM phenotype 130070), affecting connective tissue and causing skeletal deformations [70–72]. Finally, a locus for diaphyseal total area has *RAB24* and *SMIC1* as candidates. *RAB24* is one of the small GTPases of Ras-related proteins that are usually involved in intracellular trafficking. However, *RAB24* seems to participate in cell division and chromosome segregation [73]. Potential functions in bone are unknown, but *RAB24* was down-regulated in a gene expression study of stimulated mineralisation in the osteoblast-like mouse cell line MCT3-E1 [74]. The other candidate at the same QTL, *SIMC1*, regulates the protease calpain 3 in skeletal muscle [75].

### Mouse and human candidates

The number of mouse GWAS candidates for bone density that were also found as candidates in our study was restricted to four shared associations. One of them is *MRPS18A*, which has been discussed above. The others are from QTL that contain several gene expression candidates. *SSTR5* encodes one of the receptors for the hormone somatostatin. It regulates growth hormone secretion, and mutations in the gene are associated with acromegaly [76,77]. The site of action of growth hormone regulation should be the pituitary, but there is also evidence somatostatin and its receptors affect bone precursor cells [78,79]. *CACNA1H* encodes, Ca_v_3.2, a voltage gated calcium channel subunit. It is expressed in osteoblasts and chondrocytes during bone development in mice [80,81]. *GP1BA* encodes a von Willebrand protein receptor expressed on the surface of platelets. Mutations cause bleeding diseases Bernard-Soulier syndrome (OMIM phenotype number 231200 and 153670) and platelet-type von Willebrand disease (OMIM phenotype number 177820). von Willebrand factor can interact with osteoprotegrin to regulate osteoclast differentiation [82], and a transgenic *GP1BA* von Willebrand disease model has increased bone mass and reduced osteoclast activity [83]. We found no overlap between our candidate quantitative trait genes and the top associations for human bone traits in the dbGAP Association Results Browser. However, the Framingham osteoporosis study found a region around *KHDRBS3* as a potentially pleiotropic association with lumbar spine and femoral neck bone mineral density [84]. As mentioned previously in connection with the candidates, several of the genes highlighted here cause rare Mendelian diseases in humans, some of them with known skeletal phenotypes. Local cis-eQTL effects need not be as drastic as the loss-of-function mutations that cause Mendelian diseases, and could be restricted to the bone by tissue-specific regulatory elements.

### Chicken bone genetics

Based on the functional literature and the above associations, many of the genes found are plausible candidates for bone traits. However, we find a number of unexpected or unknown candidates. This is reasonable given the dearth of knowledge about the genetic machinery of complex traits in general. However, it could also be due to peculiarities of chicken bone metabolism as compared to mammals such as humans and mice. Previously unknown or poorly understood genes, coupled with the relative lack of human and mouse GWAS overlaps in our data, potentially indicate the novel aspect of chicken bone metabolism. In the female chicken femoral bone, calcium is continually removed from bone to supply the forming eggshell on a 24-hour basis throughout laying. This might require genetic mechanisms different from those in mammals. Understanding this mechanism will offer novel insights into bone metabolism.

We also find that several known genes involved in bone, including *Matrix Gla protein*, *TRAF2*, *PDZRN3* and *PDZRN4* display cis-eQTL effects. While they are not candidates for the phenotypic QTL detected in this study, this regulatory variation in bone genes suggest that there is more subtle variation in bone metabolism between wild and domestic chickens. Among the most highly expressed genes that also have an eQTL, we note *BF1* and *BF2* genes on chromosome 16, involved in immunity in the chicken, the MHC class II beta chain gene *BLB1*, the MHC *B-G* antigen, and *cathepsin S* involved in degrading protein antigens for MHC II presention. While evolution of the immune system under domestication is not the focus of this work, we note that this suggests changes to adaptive immunity.

Genetic variation affecting bone traits between wild and domestic genotypes is of interest to the evolutionary study of chicken domestication, and potentially provides insights into the molecular genetic regulation of bone mineral density. However, variation present within domestic breeds is most useful to breeders. Whether the QTL we detect segregate in contemporary domestic chickens can only be found by studies of these populations. We do at least find some replicability of the results of the F_2_ generation of the intercross used in this work [85,86]. When comparing the advanced intercross bone results to the previous F_2_ results, about half of the F_2_ QTL overlapped at least one of the F_8_ QTL. Possible reasons for differences between F_2_ and advanced intercross mapping include thee limited power to detect small-effect QTL and the larger linkage blocks causing the F_2_ study to detect the aggregate effect of several linked QTL rather than single QTL. The latter phenomenon should be more pronounced for polygenic traits with epistatic interactions, and bone traits display both a large number of loci and considerable epistasis.

Expression QTL mapping is an established genomics approach that has had several successes in quantitative trait gene identification in animals. The localisation of a QTL from a linkage study is always rough, due both to the inherently limited resolution of experimental crosses, and the statistical uncertainties of QTL mapping. Our candidate gene prioritisation builds on the overlap of 1.8 LOD drop intervals, corresponding to approximate 95% confidence intervals, between QTL and eQTL. In the next step, we test for an association between the gene expression level and the bone phenotype. We will suffer false negatives when variants act by other means than gene expression changes in adult bone tissue. Conversely, a candidate gene found might not be causative if the gene expression is correlated with the trait for some other reason, such as the gene being downstream of the causative gene in a regulatory pathway. For instance, if the bone QTL causes the proliferation of some population of bone cells, causing increases in mRNA of cell division related genes such as *CENPO* or *RAB24* and mitochondrial protein genes such as *MRPS18A*. Some of the QTL intervals have many candidate genes that are highlighted by our approach (see S2 Table). While it is possible for a QTL region to harbour multiple causative genes, false positives are likely among these candidates. In the end, conclusive evidence for a quantitative trait gene can come through experimental manipulations in cell culture or transgenic animals. Our QTL and eQTL mapping results provide compelling candidates for such experiments.

### Conclusions

In conclusion, we find several potential quantitative trait genes for bone traits in the chicken, using to our knowledge the first eQTL analysis of bone tissue in the chicken. They are supported by genetical genomics evidence in the form of QTL-eQTL overlap and trait-expression associations. While the genes are not particularly obvious candidates, some of them have connections to bone metabolism in the literature, and a handful have been associated with bone mineral density in mice or humans. Further investigation of the molecular mechanisms of these potential quantitative trait genes could reveal unexplored pathways of bone density regulation.

## Materials and Methods

### Ethics statement

The study was approved by the Regional Committee for Ethical Approval of Animal Experiments (Jordbruks verket DNR# 122–10). Birds were sacrificed by cervical dislocation and decapitation, as per the guidelines of the permit.

### Wild x domestic advanced intercross

The intercross was established from one Red Junglefowl male originating from Thailand and three White Leghorn females of the L13 line. QTL mapping has previously been performed in the F_2_ generation (see [85,86]). The intercross was maintained with approximately a hundred individuals per generation at Linköping University and expanded for QTL mapping in generation eight. Five batches of eighth generation intercross chickens were hatched and kept at the Linköping University chicken facility. They originated from 107 pairings of 122 F_7_ individuals. The chickens lived in 3 x 3 meter pens with three levels, access to perches, and food and water ad libitum. Chickens were culled when they were 212 days old. The study was approved by the Regional Committee for Ethical Approval of Animal Experiments.

### Phenotyping

Femurs were dissected out after sacrifice, the right stored frozen at -20°C for bone phenotyping while a piece cut from the left was frozen in liquid nitrogen and stored at -80°C for RNA isolation. We used peripheral quantitative computer tomography (Stratec XCT—Research SA, Stratec Medizintechnik, Germany) to measure a metaphyseal (6% of femur length) and a diaphysial (50% of femur length) cross section from each femur. We used the CORTMODE1 setting with a density threshold of >1000 mg/cm^3^ to measure cortical bone, PEELMODE2 with a threshold of 1000 mg/cm^3^ to measure endosteal area, density and bone content, PEELMODE2 with thresholds 1000 mg/cm^3^ and 150 mg/cm^3^ to measure medullary area, density and bone content, and PEELMODE2 with a threshold of 150 mg/cm^3^ to measure total area. In total, we gathered bone phenotypes from 227 male and 229 female chickens. Female chickens were also tested in egg-laying trials, as detailed in [87]. There were two fecundity trials of two weeks each. In the first, eggs were collected daily, and in the second, females were given two dummy eggs initially, and kept laid eggs. Since the second trial was closest to the time of sacrifice, we considered the number of eggs produced during this trial as a possible covariate for mapping of bone phenotypes. Females that produced no eggs at all were excluded. Bone and fecundity phenotypes from this subset of the intercross that was assayed for gene expression have previously been used to test for associations between phenotypes and gene expression of comb candidate genes; see [87,88]. Summary statistics for traits are given in S5 Table.

### QTL mapping

The chickens were genotyped for 652 SNP markers spread across the sequenced part of the chicken genome. DNA was isolated from blood samples using a standard TRIS extraction. Genotyping was performed with the Illumina Golden Gate platform at Uppsala Seq and SNP platform. We performed QTL mapping with R/qtl [89] and Haley-Knott regression using forward-selection for multiple-QTL models and pairwise scans to search for epistasis. All analyses included sex, batch and body weight as covariates. For female traits we also considered egg laying in the second fecundity trial as a possible covariate. If there was a significant association between the bone phenotype in question and the total weight of eggs produced we included the egg covariate in QTL mapping. To adjust for cryptic relatedness structure, which can be a confounder in advanced intercrosses, we applied principal component analysis on the genotype matrix, and included principal components as covariates in QTL mapping. Empirical significance thresholds were established by means of permutation tests, shuffling the phenotypes while preserving the correlation structure of the genotypes. Genomic support intervals around the QTL were formed with the 1.8 LOD drop method [90]. See S1 Data for marker informativeness values, and S2 Data for phenotype and genotype data in R/qtl format.

### Gene expression microarrays

At the time of dissection, a piece was cut from the middle of the left femur and stored in liquid nitrogen. Bone samples were disrupted with a hammer while frozen and then homogenised on a FastPrep 24 instrument using ceramic beads (Lysing matrix D, MP Biomedicals) and Tri reagent (Ambion). RNA was isolated using Tri reagent according to the manufacturer's protocol. RNA was further purified with the Fermentas GeneJet RNA purification kit (Thermo Scientific). After treatment with DNAse I, double-stranded cDNA was synthesised with a combination of Fermentas RevertAid Premium First-strand cDNA synthesis kit, DNA polymerase I, RNase H and T4 DNA polymerase (Thermo Scientific) according to protocols provided with the kit. The cDNA was labelled with the NimbleGen One Colour labelling kit (Roche) and hybridised to NimbleGen 12x135k custom gene expression microarrays. Scanning was performed with a NimbleGen microarray scanner (Roche). Subarrays were discarded due to low fluorescence intensity or visual uneven fluorescence resulting in 125 individual samples. Micoarrays were designed to cover all RefSeq and Ensembl genes as well as a database of ESTs. EST probesets were annotated by alignment to the chicken genome (version 2.1/galGal3) with BLAT [91] (see S3 Dataset). The name of each probeset used in tables contains the database accession (from RefSeq, Ensembl or the NCBI EST database) of the gene model or EST sequence used to design it. Selected gene expression data with bone and fecundity phenotypes from this subset of the intercross has previously been used for targeted genetical genomics of QTL regions for comb size; see [87,88]. Microarrays have been uploaded to ArrayExpress under accession number E-MTAB-3141.

### eQTL mapping

Expression QTL mapping was performed with Haley-Knott regression as implemented in R/qtl [89]. Local, putative cis-acting, eQTL were mapped within an interval of 100 cM around the position of the probeset. The interval was expanded to the closest flanking markers spanning at least 50 cM in each direction. Global trans-acting eQTL were mapped using the entire genetic map. A probeset was assigned a cis-eQTL when the logarithm of odds passed the cis-threshold on any markers in the window around the location of the probeset, and a trans-eQTL if it passed the (higher) trans-threshold somewhere else in the genome. 1.8 LOD drop intervals, expanded to the closest markers, were used to form confidence intervals around eQTL. Thresholds were generated by permutation separately for cis and trans associations either using the whole-genome or the 100 cM regions around probeset locations. Each iteration, the individual identities were resampled, a 100 probesets were subsampled, and the maximum LOD score from an eQTL scan of this permuted dataset was saved. The process was repeated 10000 times, and the 95th percentile LOD score was used to generate the significance threshold.

### Candidate quantitative trait genes

We used a two-phase method to search for quantitative trait genes for bone phenotypes: 1) we overlapped 1.8 LOD drop intervals from bone QTL and expression QTL; 2) tested for an association between probeset expression levels and bone traits with a regression model including body mass as covariate. Also, the same egg fecundity phenotype was included as covariate, in cases where the QTL in question was detected with a fecundity covariate. The regression between probeset expression level and bone trait included the body weight as a covariate. p-values from the t-test of the regression coefficient were adjusted by Bonferroni correction for the number of uncorrelated cis-eQTL (those where p-value for pairwise correlation test > 0.05) in the interval.

### Expression QTL colocalisation

To investigate clustering of diastal trans-eQTL, we simulated uniformly placed eQTL on an interval the size of the sequenced chicken genome and counted the maximum coverage of simulated eQTL intervals in 1000 iterations. We regard any region with coverage above the 95^th^ percentile of the simulated maximum coverage, which was 22, as an eQTL cluster.

### Comparison with human and mouse GWAS results

We compared our candidate quantitative trait genes with previously published genome-wide association studies (GWAS) in human and mouse. We used the mouse GWAS data from [35] and the human top associations catalogued in the dbGAP Association Results Browser (http://www.ncbi.nlm.nih.gov/projects/gapplusprev/sgap_plus.htm). Using Ensembl version 70, we mapped Ensembl and RefSeq probesets to Ensembl gene identifiers and to orthologous human and mouse genes. In the mouse dataset we extracted the p-values for SNPs in 200 kb windows around the start of the Ensembl gene model. The threshold for genome-wide significance used in the original study was 4 * 10^–6^. For the human associations, we exported all associations for bone mineral density in humans from dbGAP Association Result Browser with a p-value less than 10^–5^. We mapped the pair of flanking genes listed in the Association Results Browser to chicken Ensembl gene identifiers and overlapped them with the list derived from our results.

## Supporting Information

S1 Table All QTL. (XLS)

S2 Table All candidate genes. (XLS)

S3 Table All eQTL. (XLS)

S4 Table Trans-eQTL hotspots with eQTL and QTL overlapping them. (XLS)

S5 Table Summary statistics about phenotypes. (XLS)

S6 Table Overlap between F_2_ and F_8_ bone QTL. (XLS)

S7 Table Variance explained by QTL model for each trait. (XLS)

S1 Fig LOD plots of high confidence candidates. (PDF)

S2 Fig Scatterplots of high confidence candidates. (PDF)

S1 Data Phenotype and genotype data in R/qtl csv format. (CSV)

S2 Data Markers with location and informativeness. (XLS)

S3 Data Genomic locations of microarray probesets. (CSV)
