# Supplementary figures and images for: Genetic Regulation of Bone Metabolism in the Chicken: Similarities and Differences to Mammalian Systems
Source: PLoS Genet. 2015 May 29;11(5):e1005250. doi: 10.1371/journal.pgen.1005250 (PMC4449198; doi:10.1371/journal.pgen.1005250)

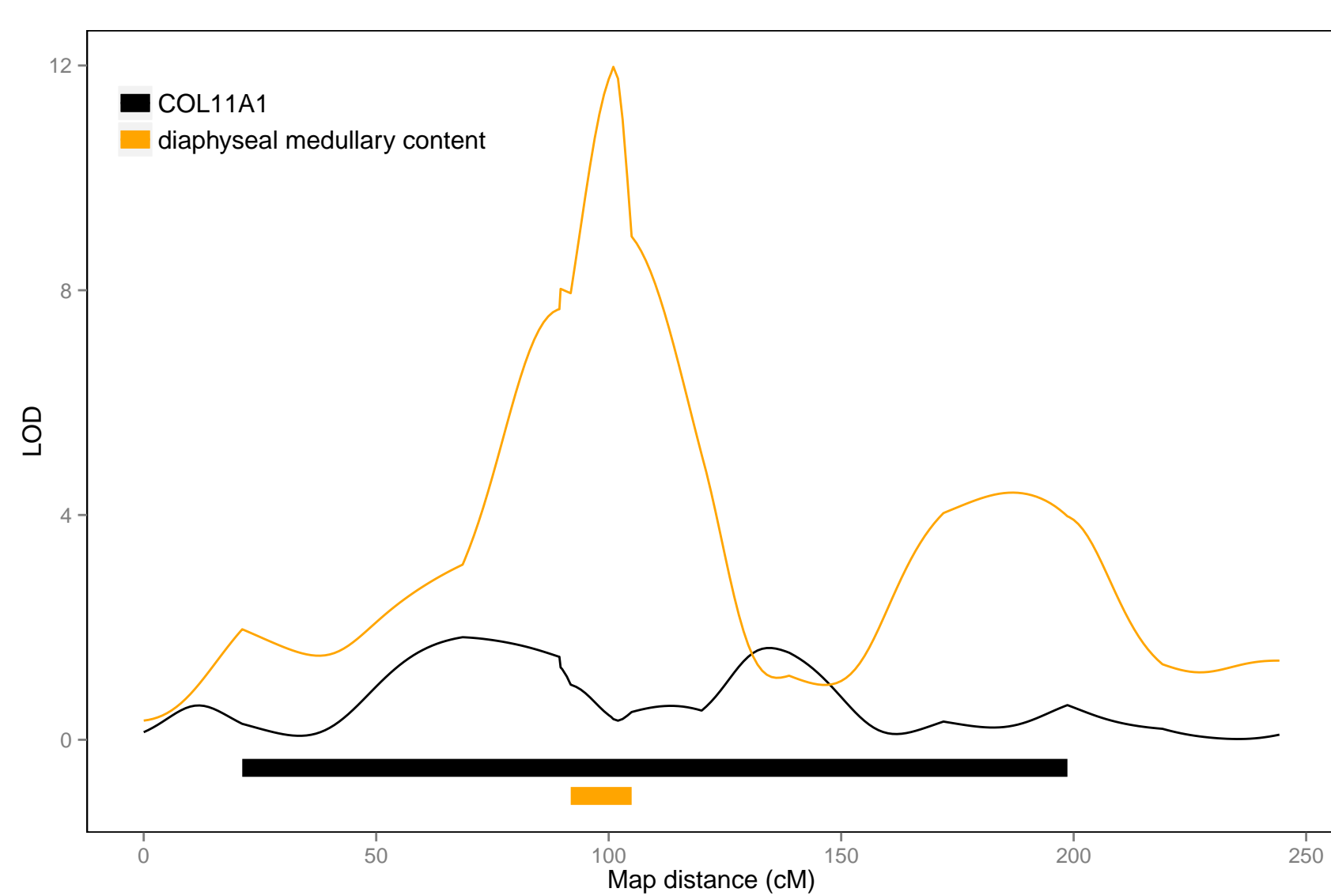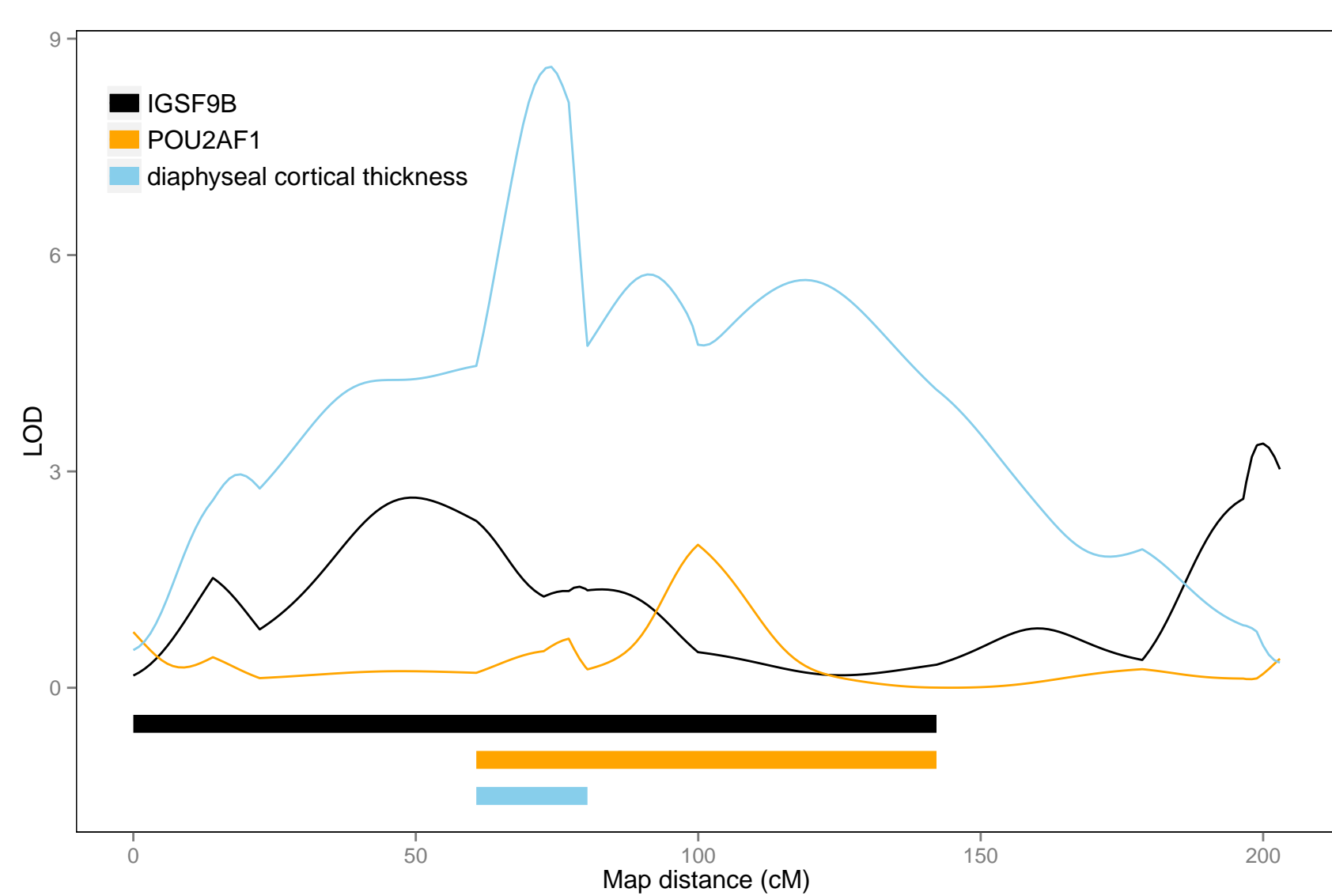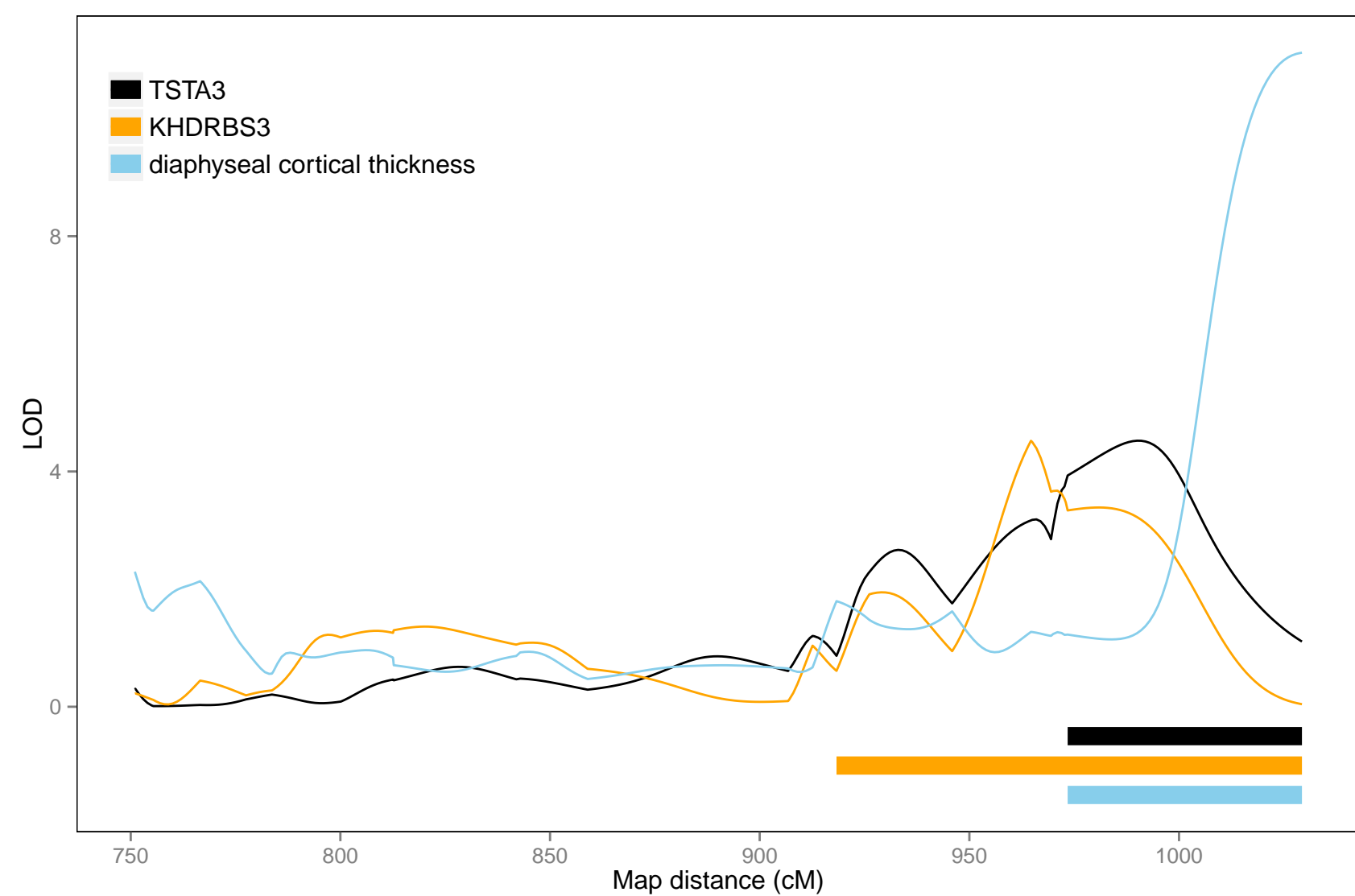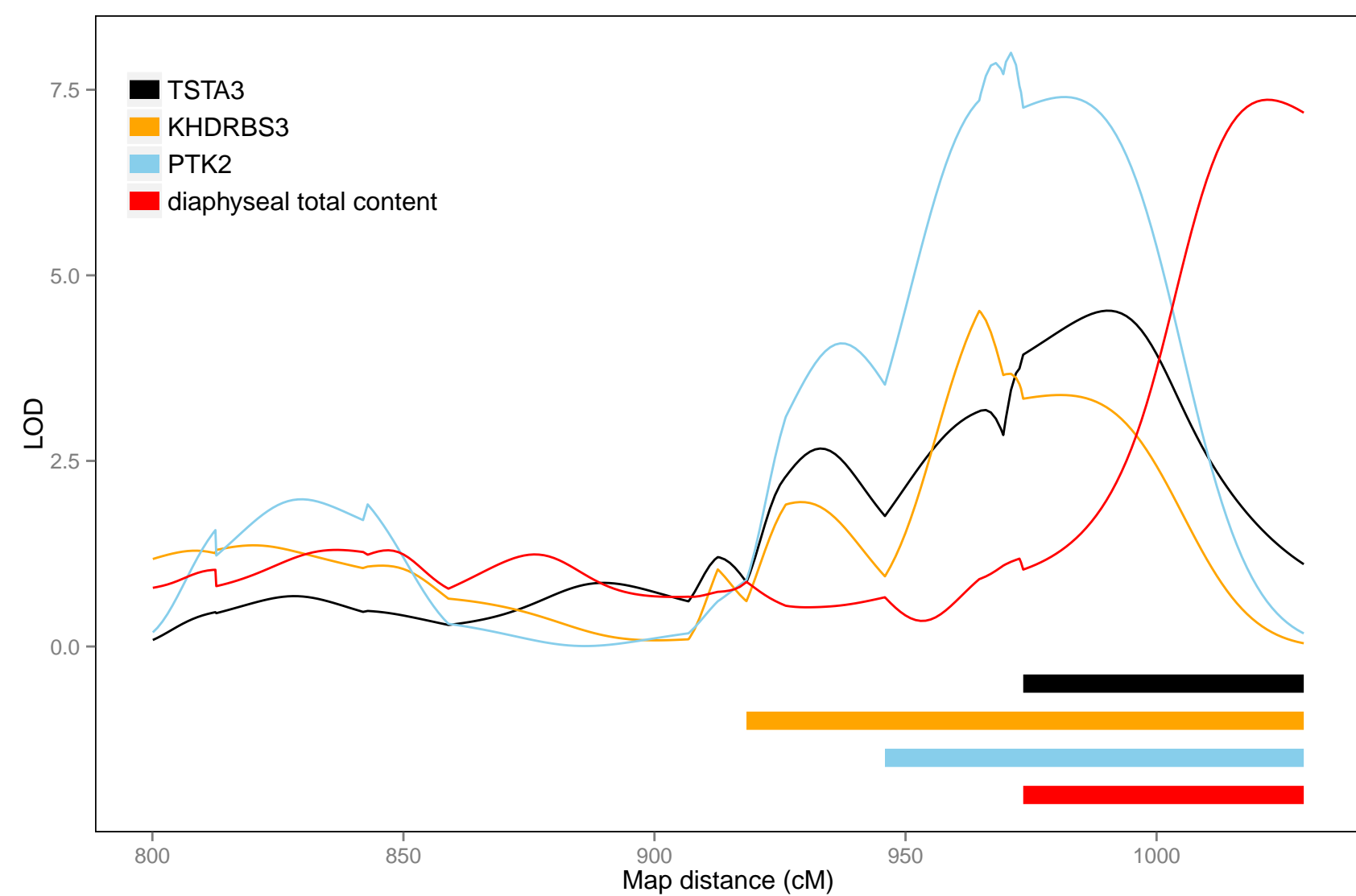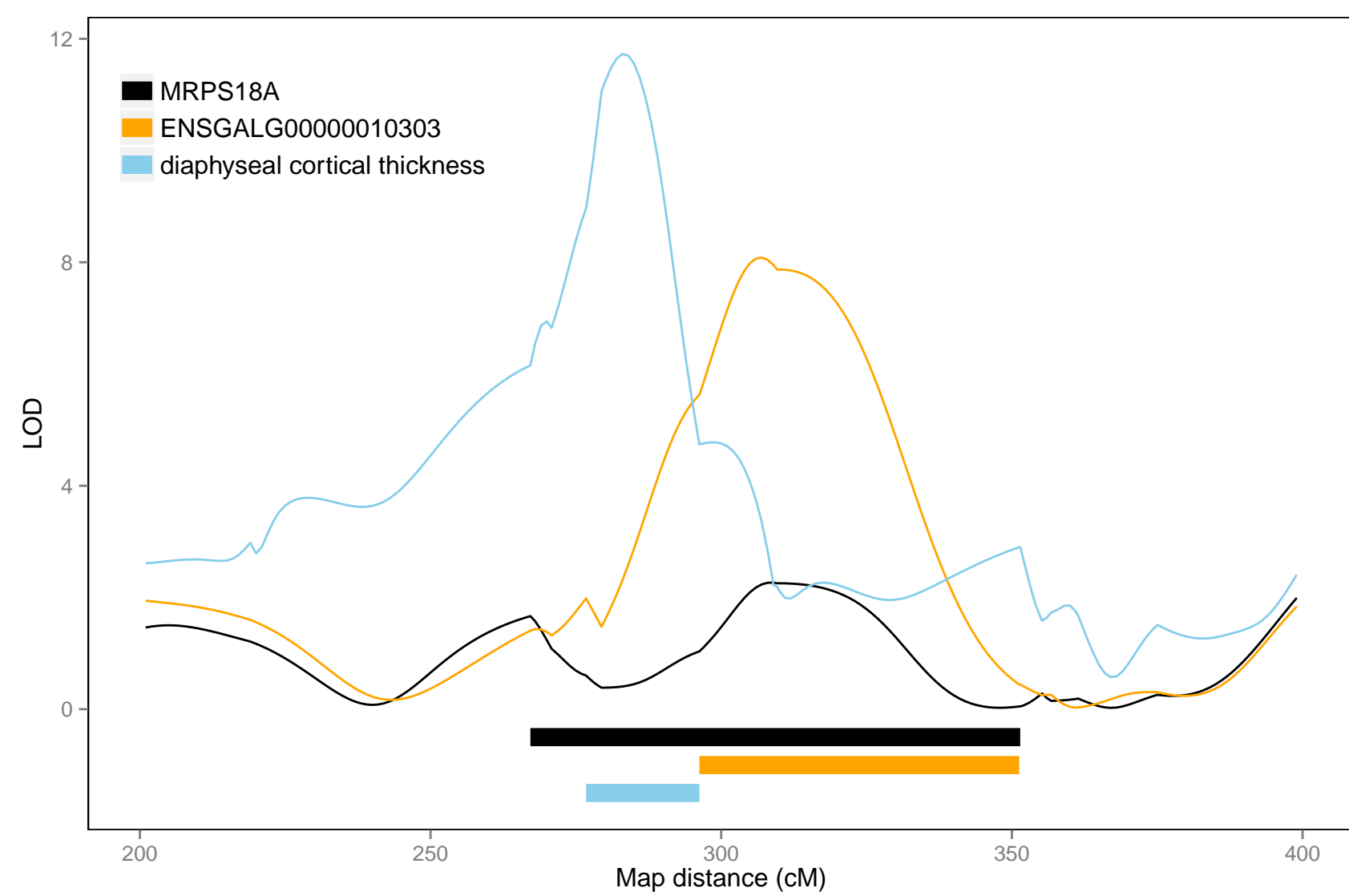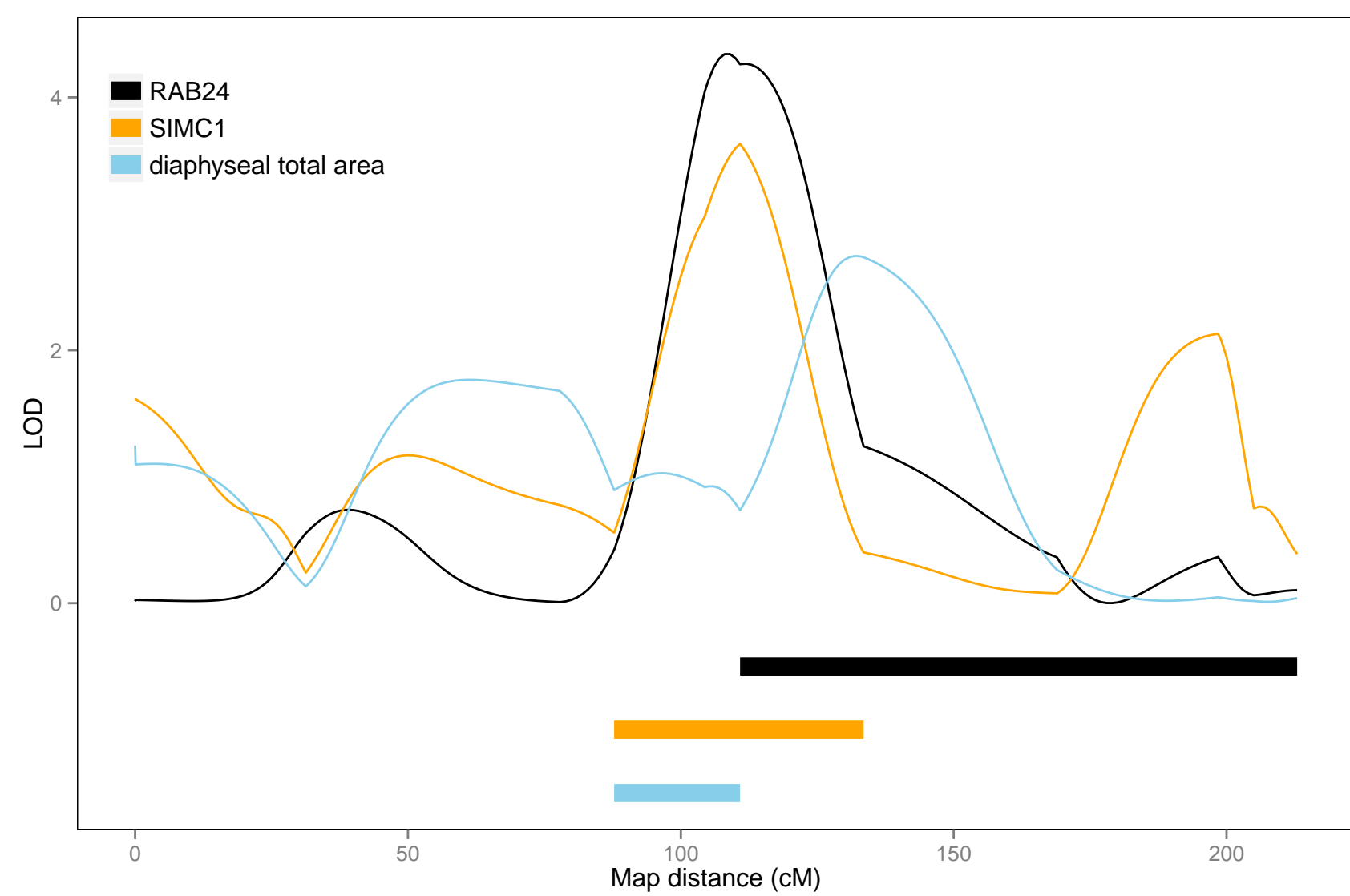

Supplement: S1 Fig — (PDF) [file pgen.1005250.s008.pdf]

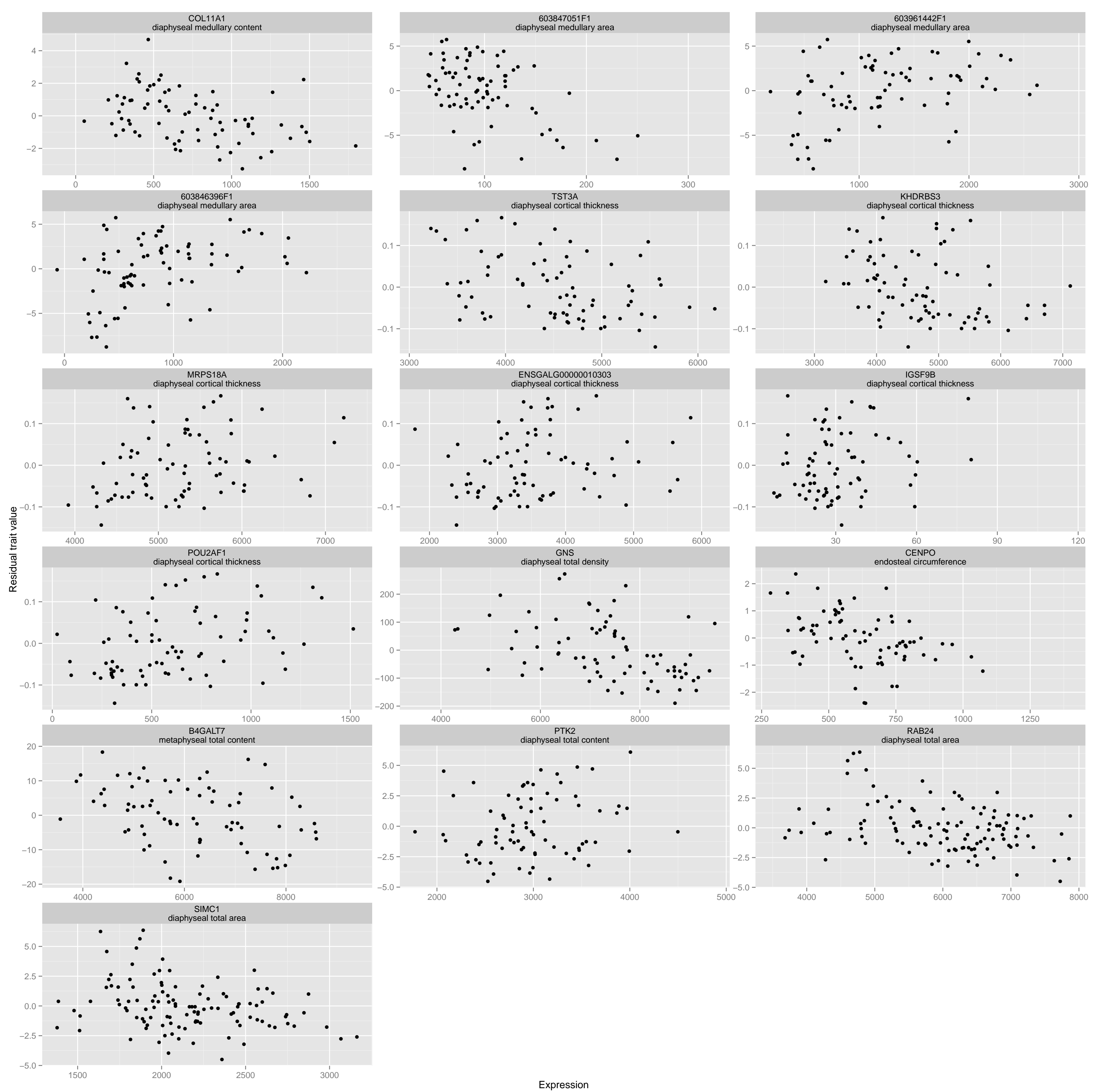

Supplement: S2 Fig — (PDF) [file pgen.1005250.s009.pdf]
